# Multimodal and Advanced Characterization of Dental Resin Composites: Insights into Beverage-Induced Degradation

**DOI:** 10.3390/jcm14124080

**Published:** 2025-06-09

**Authors:** Lucian Floare, Ramona Dumitrescu, Vanessa Bolchis, Octavia Balean, Gabriela Vlase, Titus Vlase, Iasmina-Mădălina Anghel, Carmen Opris, Ruxandra Sava-Rosianu, Vlad Tiberiu Alexa, Daniela Jumanca, Atena Galuscan

**Affiliations:** 1 Translational and Experimental Clinical Research Centre in Oral Health, Department of Preventive, Community Dentistry and Oral Health, University of Medicine and Pharmacy “Victor Babes”, 300040 Timisoara, Romania; lucian.floare@umft.ro (L.F.); dumitrescu.ramona@umft.ro (R.D.); vanessa.bolchis@umft.ro (V.B.); sava-rosianu.ruxandra@umft.ro (R.S.-R.); jumanca.daniela@umft.ro (D.J.); galuscan.atena@umft.ro (A.G.); 2 Clinic of Preventive, Community Dentistry and Oral Health, “Victor Babes” University of Medicine and Pharmacy, Eftimie Murgu Sq. no 2, 300041 Timisoara, Romania; 3 Research Centre for Thermal Analysis in Environmental Problems-ICAM, West University of Timisoara, Pestalozzi Street 16, 300115 Timisoara, Romania; gabriela.vlase@e-uvt.ro (G.V.); titus.vlase@e-uvt.ro (T.V.); 4 Department of Materials Engineering and Manufacturing, Faculty of Mechanics, Politehnica University, 300222 Timisoara, Romania; iasmina.anghel@student.upt.ro (I.-M.A.); carmen.opris@upt.ro (C.O.)

**Keywords:** dental resin composite, chemical degradation, FTIR spectroscopy, Raman spectroscopy, Vickers microhardness

## Abstract

**Background/Objectives**: Composite dental restorations are continuously exposed to dietary substances, which may compromise their structural integrity. This study aimed to assess the chemical and mechanical effects of coffee, red wine, and Coca-Cola on two widely used commercial resin composites, Herculite Ultra XRV and Omnichroma. **Methods**: Forty disk-shaped specimens (20 per material) were immersed for 10 days in the selected beverages. Changes in chemical composition were analyzed using Fourier Transform Infrared (FTIR) and Raman spectroscopy, while Vickers microhardness testing evaluated surface hardness. **Results**: FTIR and Raman analyses revealed that coffee and red wine caused the most substantial chemical degradation, particularly in carbonyl (C=O), aromatic (C=C), and siloxane (Si–O–Si) groups. Herculite XRV demonstrated higher chemical stability, while Omnichroma showed more pronounced molecular degradation. In contrast, microhardness testing indicated that Omnichroma maintained better surface hardness compared to Herculite XRV after exposure. Across all solutions, Coca-Cola induced the least effect. **Conclusions**: The tested beverages significantly affected both the chemical and mechanical properties of the resin composites. Omnichroma exhibited superior mechanical durability, while Herculite XRV showed greater resistance to chemical degradation. These results highlight the importance of material composition in restorative dentistry and support the development and selection of composites with improved resistance to acidic and staining agents to ensure long-term clinical performance.

## 1. Introduction

For centuries, dentistry has been dedicated to finding the most effective methods and materials to restore lost tooth structures or replace missing teeth. Cavities are a widespread issue in oral health, affecting 60–90% of children and 90–100% of adults [1]. Tooth decay arises when acidogenic bacteria lower the pH to 5.5–6.5, causing enamel demineralization through calcium and phosphate loss. Although saliva promotes remineralization, persistent imbalance leads to progressive carious lesions [2]. To counteract this demineralization, resin-based restorative materials—such as composites, adhesives, and primers—are employed to replace lost tooth structures. However, their common monomers, glycidyl dimethacrylate (bisGMA) and triethylene glycol dimethacrylate (TEGMA), may interfere with mineral homeostasis and promote biofilm formation [1].

Dental composites are a cornerstone of modern restorative dentistry, valued for their esthetic appeal and excellent mechanical properties [3]. Dental clinicians often prefer composite resins as the material of choice for direct restorations due to several advantages. They offer excellent esthetics, require minimal tooth preparation, and are set within a relatively short time. Additionally, they bond effectively to tooth structures, enhance overall strength, are cost-effective, and possess a versatile range of mechanical properties suitable for various dental applications [4]. Despite their advantages, composite resins pose challenges in preserving mineral balance and preventing biofilm formation, underscoring the need for improved restorative material development.

Over the decades, dental composite resins have undergone significant development, evolving from conventional macrofilled materials to modern nanohybrid and suprananospherical systems. These advancements have improved esthetics, mechanical strength, and handling properties, allowing for broader clinical indications. Parallel to material innovation, recent research has applied multi-criteria decision-making (MCDM) techniques, such as the ENTROPY-VIKOR and VIKOR-MATLAB methods, to optimize and rank restorative composites based on quantitative performance metrics—including surface hardness, degree of conversion, polymerization shrinkage, and water sorption [5,6]. These tools provide structured, data-driven support for selecting the most clinically appropriate materials under specific conditions, reflecting the increasing emphasis on both empirical evaluation and computational modeling in restorative dentistry.

Dental materials must ensure biocompatibility, bioactivity, and strong adhesion to prevent microleakage and bacterial infiltration. Given their direct clinical application, understanding their structural and chemical composition is essential, as properties can vary significantly depending on both formulation and microstructure [7].

Vibrational spectroscopic techniques such as Raman and Fourier Transform Infrared (FTIR) spectroscopy are increasingly used in dental and biomedical research due to their ability to non-invasively analyze the chemical structure of materials. These complementary methods provide distinct insights: FTIR detects changes in dipole moments, while Raman focuses on molecular polarization changes [8].

Raman approach is gaining increasing significance in biomedical research due to its minimal to non-invasive nature, non-destructive properties, high biochemical specificity, low sensitivity to water, ability to operate in the near-infrared (NIR) region, and potential for remote and in vivo applications through the use of fiber-optics [9]. Raman spectroscopy is the preferred method for the precise identification of chemical compounds, particularly the functional groups of organic molecules and their interactions with human enamel and dentin. This technique is extensively used, with numerous studies conducted to investigate the chemical properties of dental materials [8]. While both FTIR and Raman are widely used to assess the degree of conversion in composites and types of cement, they remain underutilized for broader investigations into material degradation and interaction mechanisms [7]. Raman does have limitations, such as lower sensitivity (~1%) compared to FTIR (~0.1%) and susceptibility to fluorescence interference. FT-Raman spectroscopy was developed to address these issues, enabling better analysis of impure systems like dental resins. FTIR, by contrast, requires that infrared radiation match the molecular vibration frequency and produce a change in dipole moment for absorption to occur [7].

The matrices of resin-based composites are susceptible to chemical interactions with dietary components and organic acids. Clinical exposure to such agents—via food, beverages, and saliva—can significantly affect surface hardness and roughness, accelerating the degradation and aging of restorations [10]. Previous studies by our research group have already addressed related mechanical and surface properties of the same composite materials—such as surface roughness, color stability, and morphological changes—using profilometry, scanning electron microscopy (SEM), and energy-dispersive X-ray spectroscopy (EDS) [11,12]. Hardness is one of the most critical properties of restorative materials, reflecting a surface’s resistance to penetration or deformation. It serves as a reliable indicator of various other properties, such as compressive strength, proportional limit, and ductility. International standards organizations have recognized the importance of hardness and included it in the specifications for dental materials [13].

The aim of this study is to evaluate the effects of exposure to common dietary beverages, specifically coffee, red wine, and Coca-Cola, on the chemical and mechanical properties of two widely used dental composite resins: Herculite XRV [14] and Omnichroma [15]. The investigation involves the use of Raman and Fourier Transform Infrared (FTIR) spectroscopy to analyze changes in chemical composition and molecular structure, as well as hardness testing to assess alterations in the mechanical properties of the materials. The null hypothesis of this study posits that exposure to commonly consumed beverages such as coffee, red wine, and Coca-Cola does not cause significant changes in the chemical composition or mechanical properties of the tested dental composite resins.

To provide a comprehensive understanding of how dental composites respond to common dietary challenges, this study employs a multimodal characterization strategy that integrates FTIR, Raman spectroscopy, and Vickers microhardness testing. This combination of molecular and mechanical analysis enables the correlation between beverage-induced chemical degradation and changes in surface resistance. This integrative, multimodal approach—reflected in the study title—provides a more holistic understanding of composite degradation mechanisms under realistic oral exposures.

Unlike previous studies focused on single-parameter assessments, our protocol simulates clinically relevant exposure conditions using widely consumed beverages—coffee, red wine, and Coca-Cola—thus offering clinically applicable insights for material selection and long-term restorative performance.

### Background of the Study

**Figure 1:**
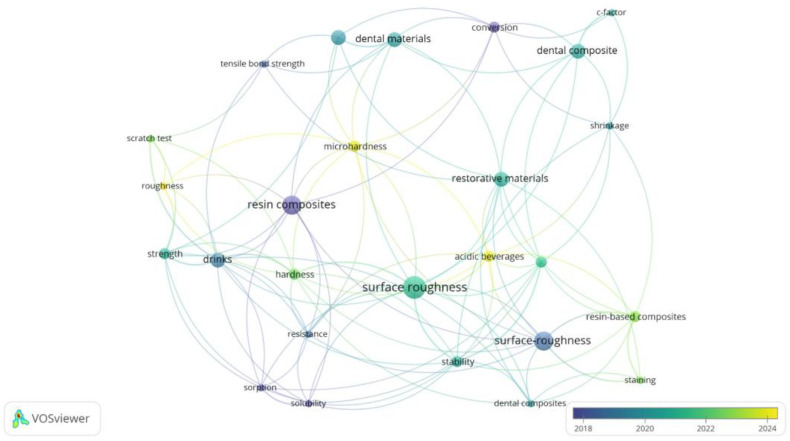
Bibliometric co-occurrence map of keywords related to resin composite degradation and characterization techniques in dental materials research (2018–2024).

To situate the current study within ongoing research trends, a bibliometric analysis was performed using VOSviewer software (version 1.6.20). The co-occurrence map in Figure 1 was generated from keywords in the recent literature on resin-based dental composites and degradation processes. The color scale reflects the average publication year, with newer topics shown in yellow and more established ones in blue–green, providing a dynamic view of thematic evolution from 2018 to 2024.

Core concepts such as “resin composites”, “surface roughness”, and “dental materials” appear as central and densely connected nodes, reaffirming their foundational role in dental materials research. These established clusters emphasize the long-standing focus on physical and mechanical characterization. At the same time, terms like “drinks”, “acidic beverages”, and “microhardness” form key connectors between surface, chemical, and strength-related nodes. This validates the interdisciplinary relevance of beverage-induced degradation, directly aligning with the present study’s multimodal approach.

Importantly, while Raman spectroscopy does not appear as a dominant term, its absence from the central map reinforces its underrepresentation in degradation-specific literature—highlighting the novelty of its integrated application alongside FTIR and microhardness in our work. Similarly, terms like “scratch test”, “conversion”, and “sorption” suggest a growing interest in correlating surface and molecular changes, which is addressed here through a combined spectroscopic-mechanical analysis.

Compared to most previous studies focusing on isolated techniques or limited beverage types, our research contributes an original integrative methodology—analyzing both chemical structure (via FTIR and Raman) and mechanical durability (via Vickers hardness)—across multiple real-life dietary challenges. By doing so, we address both compositional and functional degradation in a clinically contextualized framework.

Overall, the map supports the relevance and timeliness of this work, which resides at the intersection of traditional evaluation metrics (hardness, surface roughness) and emerging analytical depth. The multimodal design of the present study represents a methodological innovation in assessing beverage-induced composite degradation and advances the field toward more predictive, clinically oriented material assessment.

## 2. Materials and Methods

### 2.1. Materials

This in vitro study evaluated two commercially available resin-based dental composites, Herculite XRV (Kerr Corporation, Orange, CA, USA) and Omnichroma (Tokuyama Dental, Tokyo, Japan), both widely accessible in Romania. Herculite XRV is a nanohybrid resin composite selected for its excellent handling properties, high polishability, and durability. Omnichroma, a suprananospherical composite, is renowned for its universal single-shade adaptability, achieving excellent color matching by blending seamlessly with the surrounding tooth structure. Table 1 summarizes the key specifications of the materials, including their manufacturers, lot numbers, resin matrices, filler compositions, and other properties.

### 2.2. Specimen Preparation

Forty disk-shaped specimens (20 for each composite resin) were fabricated for the study. Each specimen was prepared using calibrated circular plexiglass molds with dimensions of 10 mm in diameter and 2 mm in height. A clean glass slab was placed beneath the mold to provide a stable base for accurately packing and shaping the composite material within the mold. The composite resin was packed into the molds, and a Mylar strip was applied over the surface to minimize the oxygen-inhibited layer and ensure a flat surface. A thin glass slide was then placed on top and compressed to eliminate excess material. After removing the glass slide, the specimens were polymerized using a light-curing device (Bluephase G2, Ivoclar Vivadent, Mississauga, ON, Canada) with an intensity of 1200 mW/cm^2^. The curing tip was positioned approximately 1 mm from the specimen surface to ensure even polymerization. Each specimen was cured for 40 s on both sides to achieve thorough polymerization. To ensure consistent light polymerization across all samples, the light intensity of the curing device (Bluephase G2, Ivoclar Vivadent, Mississauga, ON, Canada) was verified using a calibrated Bluephase Meter II (Ivoclar Vivadent, Inc., Mississauga, ON, Canada). Measurements were conducted prior to initiating the specimen fabrication and repeated after every 10 samples to confirm the light output remained within the manufacturer-specified intensity of 1200 mW/cm^2^. This verification protocol was adopted to minimize potential variability in the degree of conversion due to light intensity fluctuations and is consistent with previously validated methodologies employed in similar studies assessing the mechanical and chemical behavior of dental composites under standardized curing conditions [11].

After curing, the specimens were carefully removed from the molds, inspected for uniformity and absence of defects, and subjected to a standardized polishing protocol prior to immersion. This sequence—fabrication, curing, polishing, and immersion—ensured clinically relevant surface conditions and experimental consistency.

### 2.3. Polishing Procedure

After storage, all specimens underwent a standardized polishing protocol to ensure consistency. Polishing was performed for 20 s using Sof-Lex disks (3M ESPE Dental Products, St. Paul, MN, USA) in successive grit sizes: coarse (100 μm), medium (29 μm), fine (14 μm), and super fine (8 μm). A single operator polished each specimen with a low-speed handpiece operating at 15,000 rpm to ensure uniformity. Dry, intermittent pressure was applied during polishing, following the methodology described by Gonulol & Yilmaz [16]. Each specimen was polished with a new disk to avoid contamination or inconsistencies caused by disk wear.

Following polishing, specimens were ultrasonically cleaned in distilled water for 5 min and rinsed to remove residual debris. This preparation ensured smooth, uniform surfaces for reliable microhardness testing and spectroscopic analysis. Immersion in staining solutions began only after this polishing step was completed.

### 2.4. Immersion Protocol and pH Assessment of Staining Solutions

To investigate the effects of red wine, black coffee, and Coca-Cola on dental composite surfaces, specimens from each type of composite were immersed in the beverages under standardized conditions. Black coffee was prepared at a standard infusion strength using 5 g of coffee in 150 mL of boiled water (Nestlé Nespresso S.A., Vevey, Switzerland), red wine (Budureasca Clasic, Fetească Neagră, Dealu Mare, Romania) was used in its original form, and Coca-Cola (The Coca-Cola Company, Atlanta, GA, USA) was directly utilized. To ensure consistency and prevent chemical alteration due to oxidation or pH drift, each beverage (coffee, red wine, and Coca-Cola) was freshly prepared or opened daily prior to immersion. This procedure aligns with established protocols in the literature and helps maintain the reproducibility and chemical stability of the immersion environment throughout the study. The composite samples were submerged in the solutions for 20 min daily over a period of 10 consecutive days.

After each daily immersion, specimens were rinsed with distilled water, gently dried with absorbent paper, and stored in fresh distilled water at 37 °C in an incubator (Cultura Incubator 220–240 V, Ivoclar Vivadent, Bendererstrasse 2 FL-9494 Schaan, Principality of Liechtenstein) until the next cycle. Control samples were kept in distilled water at 37 °C throughout the experiment to simulate intraoral hydration and serve as a baseline. All immersion solutions (coffee, red wine, and Coca-Cola) were also maintained at 37 °C to reflect physiological conditions. This controlled environment ensured consistent hydration, minimized external variables, and allowed for reliable comparison of the chemical and mechanical effects of beverage exposure on the composite materials.

Control group specimens were not subjected to any immersion in staining or acidic solutions. Instead, they were stored in distilled water at 37 °C for the full duration of the 10-day experimental period. This method was chosen to simulate continuous intraoral hydration conditions, as described in previous studies. According to recent literature, such as the narrative review by Paolone et al. (2022) [17], distilled water remains a commonly used medium for control groups in staining experiments due to its ability to maintain material hydration without introducing extrinsic chemical or colorimetric influences. This approach ensured consistent baseline conditions against which the effects of staining beverages on chemical and mechanical properties could be accurately assessed.

Additionally, the pH of each staining solution was measured prior to the immersion process using a calibrated Milwaukee MW100 portable pH meter (Milwaukee Instruments, Inc., Rocky Mount, NC, USA) to confirm its acidic nature and ensure consistent experimental conditions. The electrode was rinsed with distilled water between measurements to maintain accuracy. The pH values recorded for the immersion solutions were 2.4 for Coca-Cola, 5.6 for coffee, and 3.5 for red wine. These results classify the beverages from strongly acidic (Coca-Cola) to moderately acidic (coffee), highlighting their potential to contribute to the surface degradation of dental composites. This procedure facilitated the standardized evaluation of the potential impact of these solutions on the surface properties of the composite specimens.

### 2.5. FTIR Spectroscopy

Fourier Transform Infrared (FTIR) spectroscopy was performed on both control and treated composite samples using the IRTracer-100 spectrophotometer (Shimadzu Corporation, Kyoto, Japan) with an ATR accessory. Data acquisition and analysis were carried out using the AIM-9000 (v.2.27) interface software, with spectra recorded over the 4000–400 cm^−1^ range at a resolution of 4 cm^−1^, averaging 20 scans per sample to ensure accuracy and consistency. This technique allowed for an in-depth examination of the chemical composition and interactions within the resin composites. FTIR spectra were recorded over a range of 4000–400 cm^−1^ with a resolution of 4 cm^−1^, providing precise detection of molecular vibrations and changes in functional groups. A diamond ATR (Attenuated Total Reflectance) accessory was employed for non-destructive sampling, ensuring consistent and reproducible results. The analysis focused on identifying structural changes induced by immersion in staining solutions by comparing the spectra of control samples with those of exposed ones. Key peaks associated with ester, carbonyl, and aromatic group vibrations were analyzed to evaluate the chemical interactions between the resin matrix and the staining agents.

### 2.6. Raman Spectroscopy

The vibrational properties of composite resins were analyzed before and after immersion in acidic solutions (coffee, red wine, and Coca-Cola) using the LabRAM Soleil™ Raman Microscope (HORIBA FRANCE SAS, Longjumeau Office, Longjumeau, France) equipped with the LabSpec 6 Spectroscopy Suite for Raman analysis. The system utilized a 532 nm laser and incorporated QScan™ lightsheet (HORIBA FRANCE SAS, Longjumeau Office, Longjumeau, France) confocal imaging for high spatial resolution, as well as SmartSampling™ (HORIBA FRANCE SAS, Longjumeau Office, Longjumeau, France) technology for rapid spectral imaging. To improve signal clarity and minimize noise, measurements were conducted with four accumulations. A 5× magnification objective lens was used to accurately focus the laser beam on the sample surface, covering a spectral range of 200 to 3200 cm^−1^ to capture a detailed vibrational profile. To ensure optimal spectral quality and avoid thermal damage to the resin, a neutral density (ND) filter at 10% (8.9 mW) was employed to regulate laser intensity. Data acquisition, processing, and spectral analysis were conducted with LabSpec 6 software, ensuring precision and consistency. Each Raman spectrum was recorded as the average of 20 scans to improve signal precision and reduce variability, ensuring consistent and reproducible results for all specimens.

### 2.7. Vickers Microhardness Analysis

**Figure 2:**
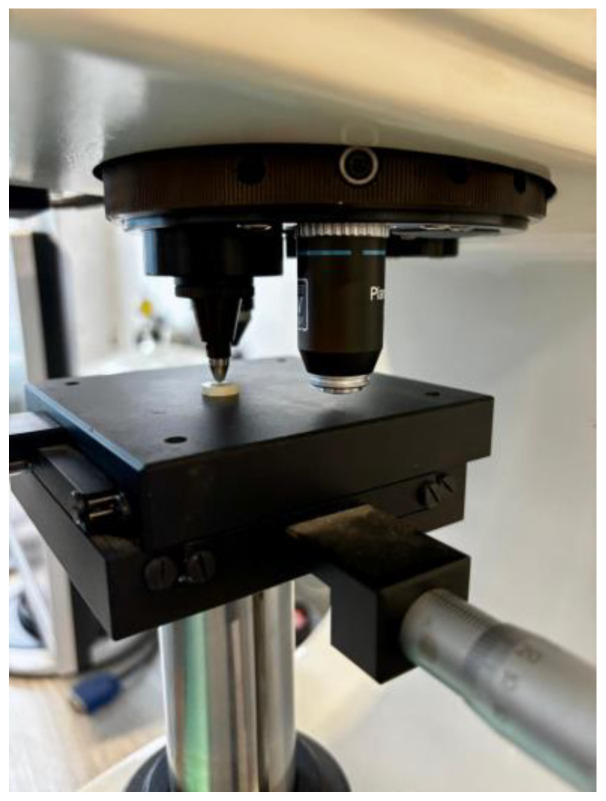
Microhardness testing setup for dental composite specimen analysis.

Microhardness was measured using the Vickers method, utilizing a diamond-shaped pyramid indenter (Figure 2). The test was performed with a 50 g load applied for 10 s, ensuring consistent and precise indentation. The samples were securely positioned during testing, and the indentation process followed the specified parameters. A Wolpert Group Micro-Vickers Hardness Tester (Model: 402MVD) was used to conduct the measurements, with results expressed in Vickers Pyramid Number (HV), a standard unit for hardness evaluation. To improve measurement precision and account for surface heterogeneity, three separate indentations were made on each specimen at distinct locations, in accordance with standard Vickers microhardness testing procedures. The average of these three values was used to determine the final microhardness for each sample, ensuring a more reliable and representative evaluation of the composite’s surface hardness by reducing the impact of localized variation. The Vickers test offers several advantages over other hardness testing methods, as its calculations are independent of the size of the indenter, making it suitable for a wide range of materials with varying hardness levels [18]. The Vickers microhardness values were calculated using the following formula:

H*v* = 1.8544*Pd*/2

where H*v* represents Vickers micro-hardness, *P* is the indentation load, and *d* is the length of the diagonal of the indentation.

### 2.8. Statistical Analysis

The microhardness data obtained before and after immersion were analyzed to calculate the percentage change, mean percentage change, and standard deviation. To determine whether the differences in Vickers microhardness before and after immersion in the staining solutions were statistically significant, paired *t*-tests were performed. A significance level of *p* < 0.05 was applied. Statistical analyses were carried out using SPSS software, version 23 (IBM Corporation, Armonk, NY, USA).

## 3. Results

**Figure 3:**
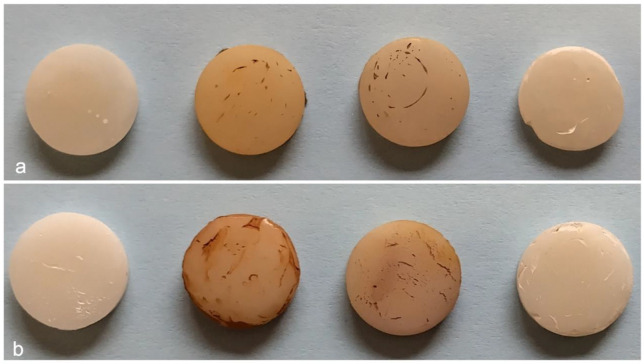
Macrophotographic analysis of resin composite specimens after 10-day beverage immersion. (**a**) Omnichroma specimens; (**b**) Herculite XRV specimens. From left to right in each row: control, coffee, red wine, and Coca-Cola.

Figure 3 illustrates the visual changes observed in the composite specimens after 10 days of daily immersion in staining beverages. In Figure 3a, Omnichroma specimens are shown, while Figure 3b presents Herculite XRV samples. From left to right: control (unexposed), coffee, red wine, and Coca-Cola. Both materials exhibited visible discoloration following exposure, with the most pronounced staining seen in the coffee and red wine groups. The control samples retained their original appearance, and Coca-Cola caused minimal visual changes. Overall, Herculite XRV showed more noticeable surface staining, particularly after coffee immersion, compared to Omnichroma.

### 3.1. FTIR Spectroscopy Results

The FTIR spectra of Herculite Ultra XRV composite resin reveal substantial chemical modifications after immersion in coffee, red wine, and Coca-Cola, compared to the control sample, which represents the baseline chemical structure of the material.

**Figure 4:**
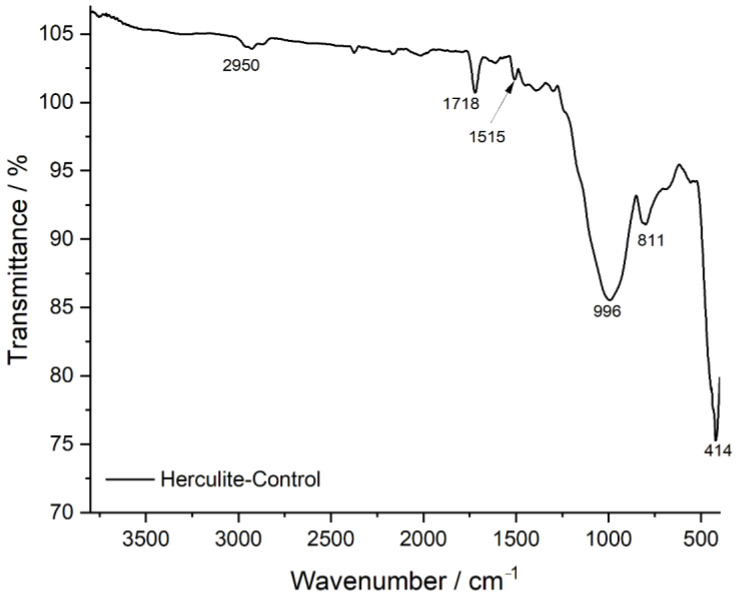
FTIR spectrum of Herculite-control: baseline chemical composition.

The FTIR transmittance spectrum of Herculite Ultra XRV (Control sample) provides a reference for the composite resin’s chemical composition before exposure to external agents. The characteristic peaks observed at specific wavenumbers (cm^−1^) correspond to key functional groups within the resin matrix. The strong absorption at 2950 cm^−1^ is attributed to C-H stretching vibrations, primarily from methyl (-CH_3_) and methylene (-CH_2_-) groups in the polymer backbone. The peak at 1718 cm^−1^ corresponds to the C=O (carbonyl) stretching vibration, which is a defining feature of the resin’s ester or urethane dimethacrylate (UDMA) structure. The signal at 1515 cm^−1^ is associated with aromatic C=C stretching, indicative of aromatic rings present in the Bis-GMA or other resin monomers. The peak at 996 cm^−1^ corresponds to C-O stretching, typically linked to ether or ester functionalities, contributing to the polymer’s cross-linked structure. The bands at 811 cm^−1^ and 414 cm^−1^ correspond to Si-O stretching vibrations, indicating the presence of silica-based inorganic fillers that contribute to the composite’s mechanical properties. The presence and intensity of these peaks establish the baseline chemical structure of the composite resin, against which the effects of exposure to acidic or staining agents can be compared in subsequent analyses (Figure 4).

Among the tested solutions, coffee exposure (blue spectrum) induced the most pronounced spectral shifts, particularly in the 1000–1700 cm^−1^ range, where significant alterations were observed in carbonyl (C=O), aromatic (C=C), and silane coupling groups (Si–O–Si). The increased intensity of absorption bands in this region suggests potential chemical interactions between coffee’s tannins and acidic compounds with the organic matrix, leading to depolymerization or degradation of the resin’s structural network. Additionally, modifications in the 1030–1080 cm^−1^ region indicate potential hydrolytic effects, suggesting that coffee may contribute to the breakdown of the silane coupling agent, weakening the bond between the filler and polymer matrix.

Red wine exposure (green spectrum) led to moderate spectral alterations, particularly in the 900–1400 cm^−1^ region, where shifts in intensity suggest significant polyphenolic interactions with the resin composite. The presence of phenolic compounds in red wine can contribute to chemical reactions with the ester functional groups within the polymer matrix, leading to ester hydrolysis and potential softening of the material’s structure. Furthermore, the spectral changes observed near 1450 cm^−1^ and 1740 cm^−1^ suggest interactions with aromatic and carbonyl groups, further indicating possible chemical degradation or cross-linking alterations in the composite structure.

**Figure 5:**
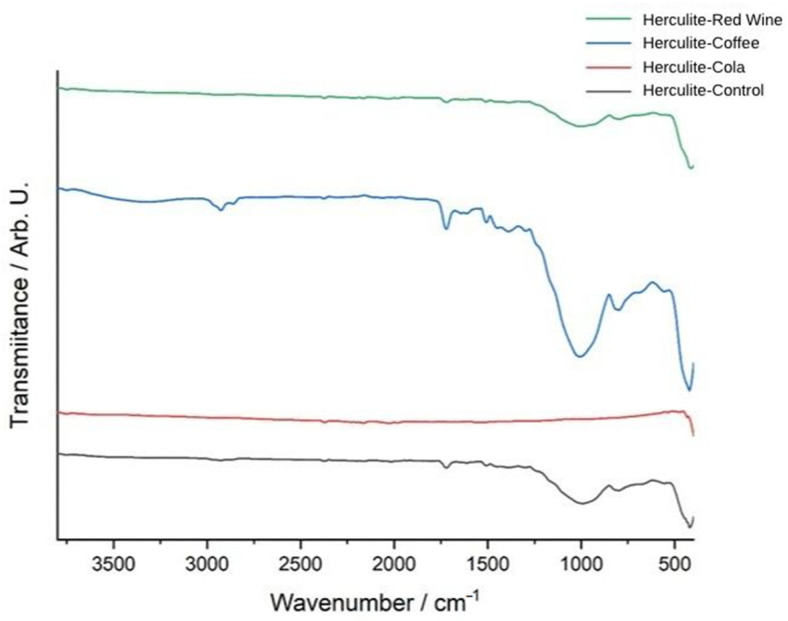
FTIR spectra of Herculite resin: control and post-immersion in coffee, red wine, and Coca-Cola.

In contrast, Coca-Cola exposure (red spectrum) resulted in minimal but noticeable spectral changes, with only slight variations near 1700 cm^−1^ and 1200 cm^−1^, indicating mild hydrolytic degradation due to acidic components present in the beverage. The lower degree of spectral shifts compared to coffee and red wine suggests that Coca-Cola’s effect on the polymer matrix is relatively weaker, primarily due to its lower polyphenol content and absence of tannins, which play a significant role in composite degradation. However, the observed slight alterations in ester and aromatic regions indicate that the low pH of Coca-Cola (around 2.4) still contributes to minor chemical changes, though not as extensively as coffee and red wine. Overall, these findings suggest that coffee had the most pronounced impact on the chemical structure of the composite resin, followed by red wine, while Coca-Cola exhibited the least effect (Figure 5).

**Figure 6:**
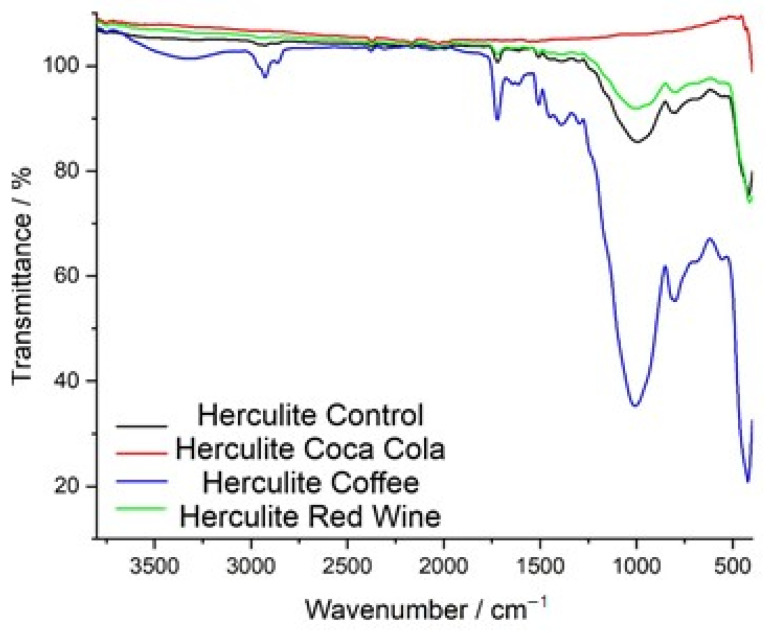
Comparative FTIR spectra of Herculite resin: control vs. immersion in Coca-Cola, coffee, and red wine.

The FTIR transmittance spectra of Herculite Ultra XRV composite resin reveal significant chemical modifications after exposure to coffee, red wine, and Coca-Cola, compared to the control sample. The control spectrum (black) represents the baseline chemical composition of the resin. The coffee-exposed sample (blue spectrum) shows the most pronounced changes, particularly in the 1000–1700 cm^−1^ range, with peaks associated with carbonyl, aromatic, and ester functional groups displaying significant intensity variations and shifts. The red wine-treated sample (green spectrum) exhibits moderate spectral changes, especially in the 900–1500 cm^−1^ region, reflecting alterations in specific functional group regions. The Coca-Cola-exposed sample (red spectrum) shows the least impact, with minor shifts in transmittance, particularly in regions corresponding to ester and aromatic groups. These results demonstrate varying degrees of chemical changes in the resin depending on the type of beverage exposure (Figure 6).

**Figure 7:**
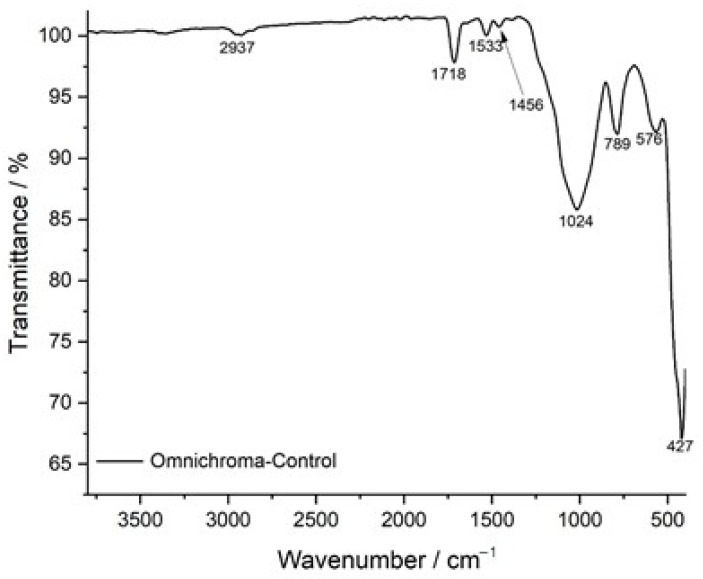
FTIR spectrum of Omnichroma-control: baseline chemical composition.

The FTIR transmittance spectrum of the control sample of Omnichroma composite resin serves as a reference for its chemical composition and structural integrity before immersion in staining solutions. The spectrum displays well-defined peaks characteristic of the composite’s polymer matrix and filler components. The broad absorption band at 2937 cm^−1^ corresponds to C-H stretching vibrations, indicative of the resin’s organic backbone, which primarily consists of UDMA and TEGDMA monomers. The strong peak at 1718 cm^−1^ is attributed to C=O (carbonyl) stretching, representing the ester functionalities within the polymer network, which are crucial for maintaining cross-linking stability and structural integrity. The presence of a distinct peak at 1533 cm^−1^ corresponds to C=C stretching in aromatic rings, confirming the presence of rigid monomer structures that contribute to the mechanical strength and wear resistance of the composite. Additionally, the band at 1456 cm^−1^ represents C-H bending vibrations, associated with the methylene (-CH_2_-) groups, which enhance the flexibility and toughness of the polymer network. The peak at 1024 cm^−1^ is associated with C-O stretching, which plays a significant role in the polymer backbone, contributing to the composite’s chemical durability. The observed peaks at 789 cm^−1^ and 576 cm^−1^ correspond to Si-O-Si stretching vibrations, confirming the presence of silica-based fillers, which enhance the mechanical reinforcement, wear resistance, and longevity of the composite material. The lowest-frequency peak at 427 cm^−1^ is attributed to inorganic filler-related vibrations, further supporting the presence of reinforcing particles within the polymer matrix. The well-defined nature of these peaks indicates a stable and structurally intact composite, providing a critical baseline for evaluating potential degradation, molecular changes, and chemical interactions following exposure to acidic and staining solutions (Figure 7).

**Figure 8:**
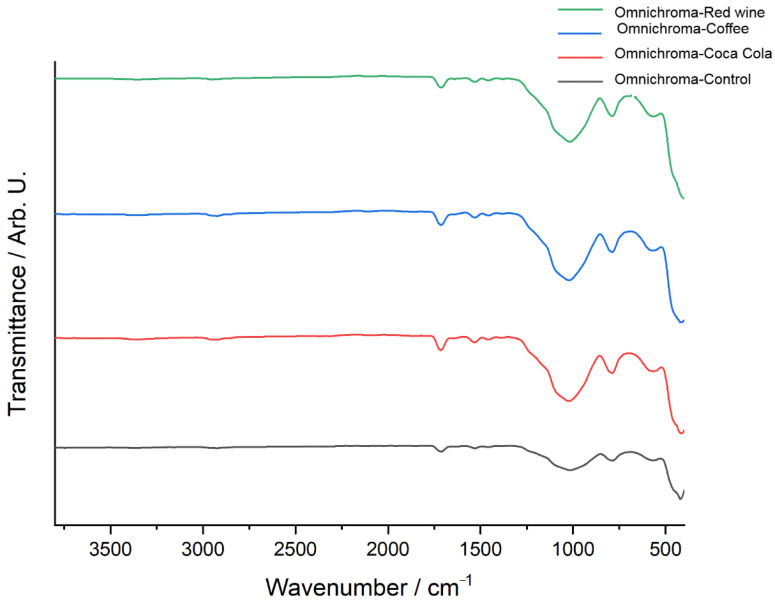
FTIR spectra of Omnichroma: control vs. immersion in coffee, red wine, and Coca-Cola.

Figure 8 presents the FTIR spectra of Omnichroma composite resin after immersion in red wine, coffee, and Coca-Cola, compared to the control sample, highlighting the chemical modifications induced by each solution. The control spectrum (black) serves as a reference, exhibiting well-defined peaks corresponding to the polymer matrix and filler components. The red wine-exposed spectrum (green) shows the most pronounced deviations, particularly in the 900–1600 cm^−1^ region, where significant shifts in C=O (carbonyl), C=C (aromatic), and Si–O–Si (silane coupling) functional groups suggest polymer degradation, ester hydrolysis, and weakening of the filler-matrix interface. The coffee-exposed spectrum (blue) also exhibits substantial alterations, with notable changes in the 1000–1700 cm^−1^ region, where shifts in carbonyl, aromatic, and siloxane bonds suggest depolymerization and chemical softening of the resin matrix due to the high polyphenolic content of coffee. Meanwhile, the Coca-Cola-exposed spectrum (red) displays less prominent spectral variations, with only minor transmittance shifts around 1700 cm^−1^ and 1200 cm^−1^, indicating mild hydrolytic degradation primarily due to the acidic nature of Coca-Cola rather than a strong interaction with the polymer structure. Overall, the FTIR analysis suggests that red wine had the most pronounced impact on Omnichroma’s chemical structure, followed by coffee, while Coca-Cola exhibited the least effect, reinforcing the role of acidic and polyphenolic compounds in composite resin degradation.

**Figure 9:**
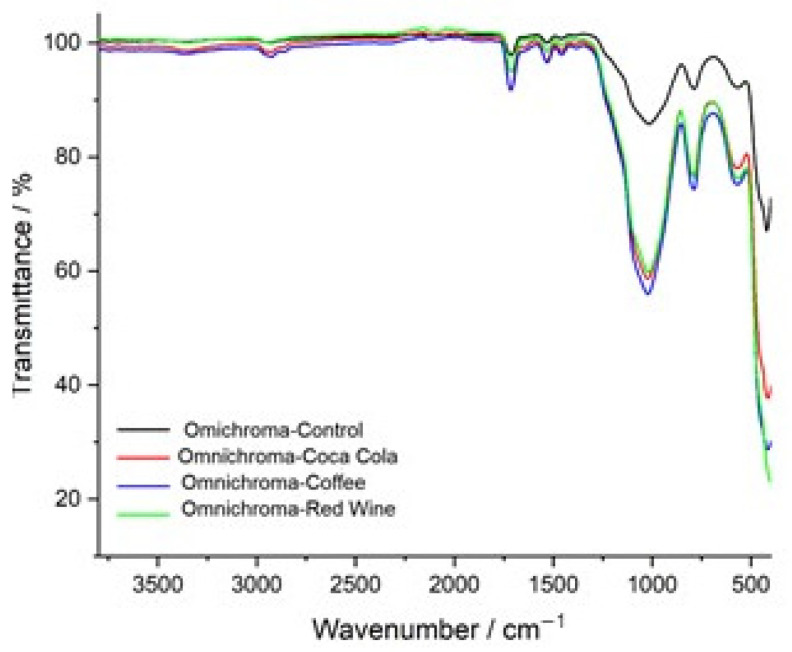
FTIR spectra comparison of Omnichroma: control vs. exposure to coffee, red wine, and Coca-Cola.

The FTIR transmittance spectra of Omnichroma composite resin before and after immersion in red wine, coffee, and Coca-Cola highlight chemical changes caused by each staining solution. The control spectrum (black) shows well-defined peaks for the polymer matrix and filler materials. Red wine (green) and coffee (blue) produce the most significant changes, particularly in the 1000–1700 cm^−1^ range, where shifts in carbonyl (C=O), aromatic (C=C), and siloxane (Si–O–Si) bonds indicate structural modifications. Red wine causes peak broadening and intensity reductions due to polyphenol-induced degradation, while coffee shows peak variations linked to tannin interactions and matrix disintegration. Coca-Cola (red) has a milder effect, with slight shifts and reductions indicating limited hydrolytic degradation. Overall, red wine and coffee significantly compromise Omnichroma’s structural integrity, while Coca-Cola has a weaker impact (Figure 9).

### 3.2. Raman Spectroscopy Results

**Figure 10:**
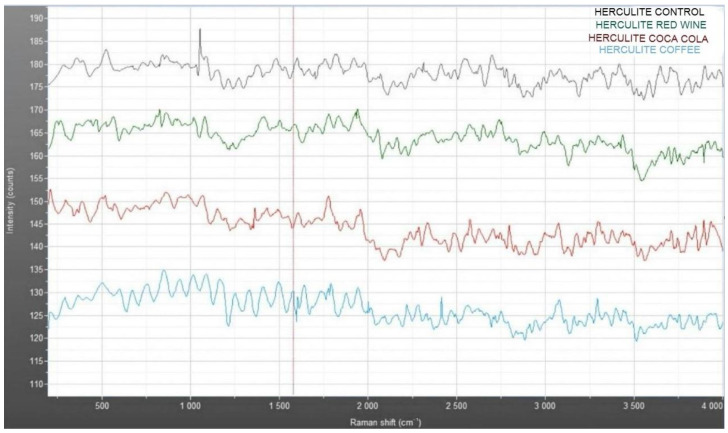
Raman spectra of Herculite XRV: comparative analysis of control and samples immersed in coffee, Coca-Cola, and red wine.

The Raman spectra in Figure 10 compare the vibrational characteristics of Herculite composite resin under different conditions: control (blue), red wine immersion (green), Coca-Cola immersion (red), and coffee immersion (light blue). The control sample exhibits distinct and stable intensity peaks across the Raman shift range, serving as a baseline for structural integrity. In contrast, the spectra for samples exposed to red wine, Coca-Cola, and coffee reveal noticeable intensity reductions and variations in peak positions, particularly in the 500–1500 cm^−1^ region. These shifts suggest alterations in the chemical structure and bonding within the resin matrix due to interaction with the staining solutions. Among the immersed samples, the red wine-treated specimen (green) displays moderate changes, while Coca-Cola (red) shows less pronounced deviations compared to coffee (light blue), which exhibits the most significant decrease in intensity, indicating potential degradation or structural modification.

**Figure 11:**
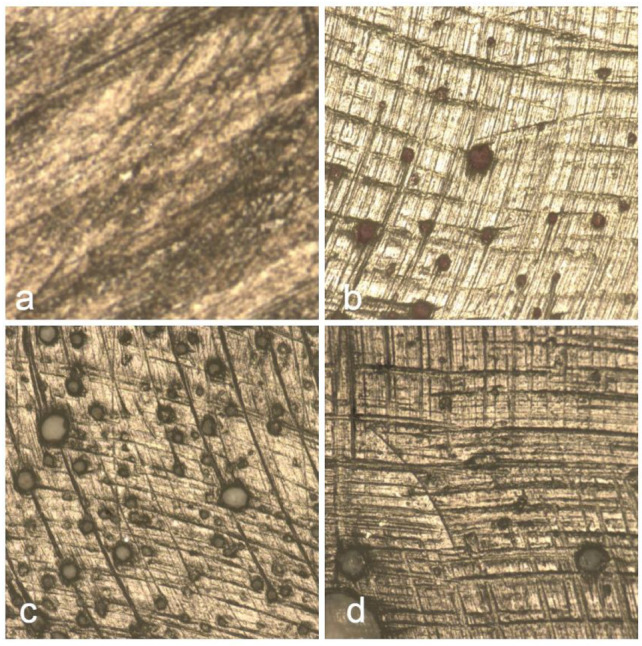
Surface morphology of Herculite composite following exposure to coffee (**a**), red wine (**b**), and Coca-Cola (**c**), with control sample (**d**) for comparison.

Figure 11 displays the surface morphology of the Herculite composite resin after immersion in various beverages, highlighting the topographical changes induced by each solution. Image (a) shows the surface after coffee exposure, where irregular striations and darkened regions suggest surface roughening and pigment penetration. In (b), red wine-treated samples reveal scattered dark deposits, likely due to polyphenolic compounds adhering to the surface. Image (c), representing Coca-Cola exposure, presents numerous rounded micro-defects possibly associated with acidic erosion. The control sample (d) maintains a relatively uniform texture, with minimal surface disruptions, serving as a reference for untreated composite integrity.

**Figure 12:**
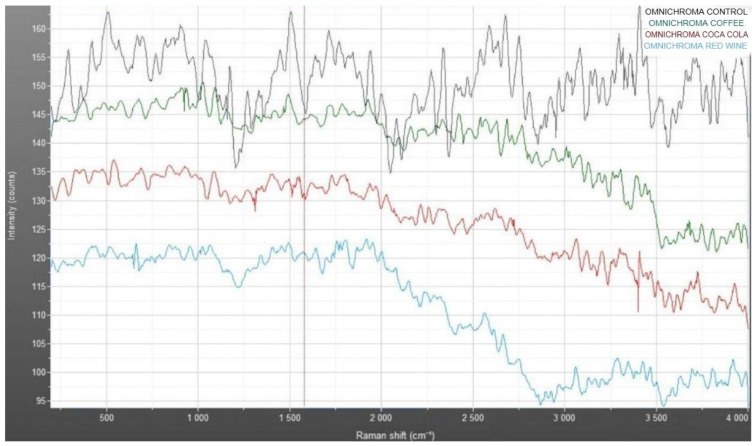
Raman spectra of Omnichroma: comparative analysis of control and samples immersed in coffee, Coca-Cola, and red wine.

The Raman spectra presented in Figure 12 compare the vibrational profiles of Omnichroma composite resin under different conditions: control (blue), coffee immersion (green), Coca-Cola immersion (red), and red wine immersion (light blue). The control sample exhibits consistent and well-defined intensity peaks across the Raman shift range, serving as the baseline for the material’s structural integrity. In comparison, the immersed samples show varying degrees of spectral changes, particularly in the 500–1500 cm^−1^ region, where shifts and intensity reductions are observed. Coffee (green) and Coca-Cola (red) display moderate alterations in peak positions and intensity, indicating chemical interactions with the resin matrix. Red wine immersion (light blue) shows the most pronounced spectral changes, characterized by broader peaks and decreased intensity, suggesting significant structural modifications. These variations reflect the differential impact of staining solutions on the molecular structure of the composite material.

**Figure 13:**
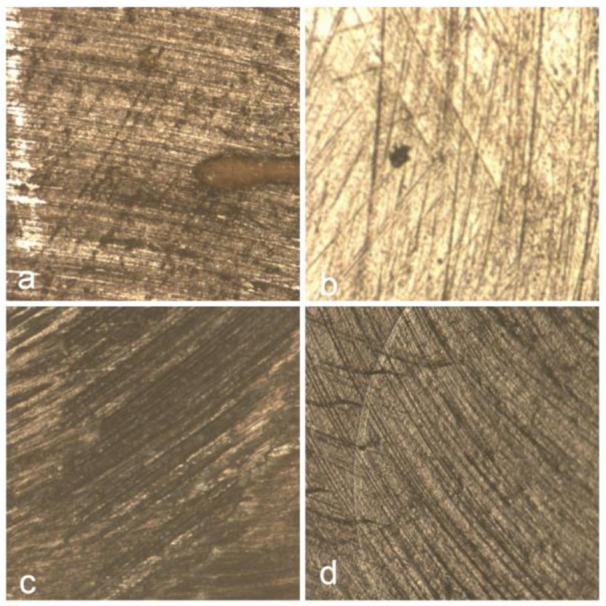
Surface morphology of Omnichroma composite following exposure to coffee (**a**), red wine (**b**), and Coca-Cola (**c**), with control sample (**d**) for comparison.

Figure 13 illustrates the surface morphology of the Omnichroma composite resin following immersion in various beverages. Figure 13a shows the surface after coffee exposure, revealing irregular striations and dark discoloration, indicating significant surface staining and potential matrix degradation. In Figure 13b, the red wine-treated sample displays a smoother surface with fewer but noticeable pigment deposits, suggesting moderate interaction with the resin surface. Figure 13c, corresponding to Coca-Cola exposure, presents less pronounced changes, with a relatively uniform texture and subtle alterations, indicating a minimal erosive impact. The control sample Figure 13d maintains a homogenous and well-preserved surface, demonstrating the material’s baseline condition in the absence of staining or acidic challenge.

These spectroscopic findings were further supported by quantitative microhardness measurements, providing a numerical assessment of surface degradation and facilitating a direct correlation between molecular-level changes and mechanical performance.

### 3.3. Vickers Microhardness Results

The microhardness of the evaluated composite materials was determined using the Vickers method. For each material, individual measurements and their mean values, along with statistical descriptors, are presented below. The hardness results are expressed as Vickers Pyramid Number (HV) and summarized in Table 2.

**Figure 14:**
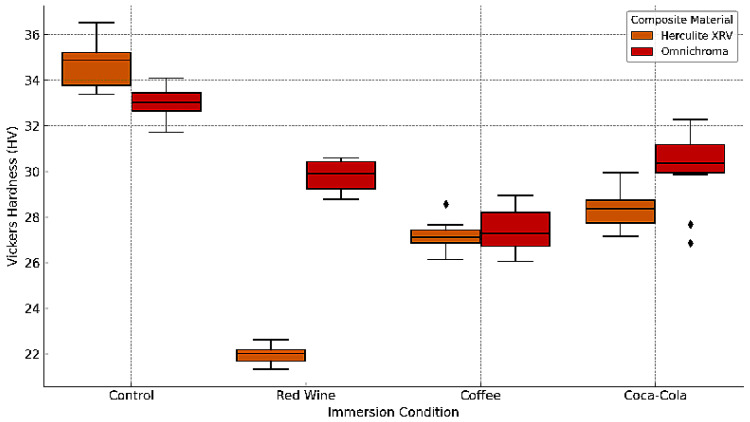
Box plot of Vickers microhardness for Herculite XRV and Omnichroma composites after immersion in coffee, red wine, Coca-Cola, and control conditions. The diamond symbols denote individual outlier measurements that fall outside the interquartile range.

The Vickers microhardness of two resin composite materials, Herculite XRV and Omnichroma, was assessed to determine the effects of 10-day immersion in coffee, red wine, and Coca-Cola, compared to control samples stored in distilled water. The data reveal a clear trend of reduced surface hardness across all experimental groups following exposure to staining solutions. Herculite XRV exhibited the highest mean hardness in the control group (34.10 ± 1.53), with a substantial decrease to 22.45 ± 0.58 after red wine exposure, followed by 27.35 ± 0.84 in the coffee group, and 28.60 ± 0.73 for Coca-Cola. Similarly, Omnichroma showed a mean control hardness of 33.20 ± 0.84, which declined to 29.60 ± 0.96 after red wine immersion, 27.42 ± 1.14 in the coffee group, and 30.26 ± 1.30 following Coca-Cola exposure (Figure 14).

Statistical analysis using paired t-tests revealed significant differences in the Vickers hardness of both Herculite XRV and Omnichroma composites following immersion in staining beverages compared to their respective control samples. For Herculite XRV, the most notable decrease in surface hardness was observed after red wine exposure (M = 22.45, SD = 0.58), compared to the control (M = 34.10, SD = 1.53), with a significant result (t(18) = 12.761, *p* < 0.01). Coffee also led to a significant reduction (M = 27.35, SD = 0.84, t(18) = 8.231, *p* < 0.01), while Coca-Cola had a slightly milder, but still significant impact (M = 28.60, SD = 0.73, t(18) = 7.234, *p* < 0.01).

For Omnichroma, the highest surface degradation was noted after coffee immersion (M = 27.42, SD = 1.14), showing a significant drop compared to the control (M = 33.20, SD = 0.84, t(18) = 8.674, *p* < 0.01). Red wine also produced a significant effect (M = 29.60, SD = 0.96, t(18) = 7.902, *p* < 0.01), followed by Coca-Cola (M = 30.26, SD = 1.30, t(18) = 5.785, *p* < 0.01).

### 3.4. Correlation Between Chemical Degradation and Mechanical Properties

**Figure 15:**
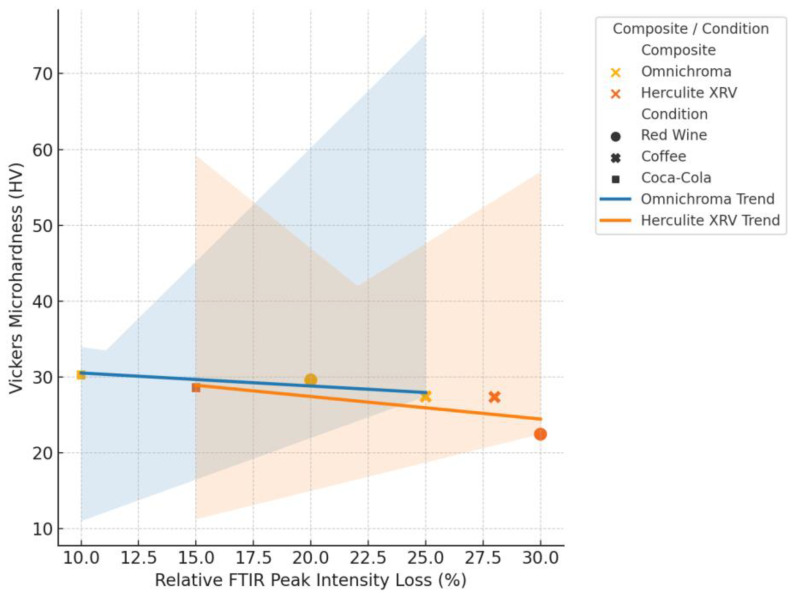
Correlation between FTIR peak intensity loss and Vickers microhardness reduction in resin composites.

To further explore the relationship between molecular and mechanical changes in resin composites, a correlation analysis was performed between the relative loss of FTIR peak intensity and the percentage reduction in Vickers microhardness following beverage exposure. In this expanded analysis (Figure 15), data were disaggregated by material type—Omnichroma and Herculite XRV—to assess whether degradation mechanisms varied across composite formulations. The resulting scatterplot shows two distinct linear trends, one for each composite. In both cases, greater spectroscopic alterations were associated with more substantial surface hardness loss, confirming that beverage-induced chemical degradation—particularly involving ester and siloxane functional groups—can compromise filler–matrix stability. Notably, Herculite XRV exhibited a slightly stronger correlation, suggesting a more predictable mechanical response to molecular changes, while Omnichroma showed a more dispersed pattern, potentially due to its suprananospherical filler technology.

## 4. Discussion

Monochrome composite resins are increasingly favored in clinical practice due to their ability to simplify shade selection, minimize waste, and deliver excellent esthetic outcomes, while nanohybrid composites are valued for their superior mechanical properties and durability. The balance between these attributes is critical for achieving successful restorations, as resin-based composites must exhibit sufficient physical, mechanical, and biological resilience to withstand the erosive and abrasive challenges of the oral environment. The results of this study reject the null hypothesis, demonstrating that exposure to coffee, red wine, and Coca-Cola significantly alters both the chemical composition and mechanical properties of the tested dental composite resins, with variations observed based on the material and type of beverage.

In our study, the FTIR analysis of Herculite Ultra XRV and Omnichroma composite resins following exposure to coffee, red wine, and Coca-Cola revealed distinct differences in their chemical stability and degradation patterns. Coffee and red wine caused the most significant chemical modifications in both materials, particularly in the 1000–1700 cm^−1^ spectral region, where functional groups such as carbonyl (C=O), aromatic (C=C), and siloxane (Si–O–Si) exhibited marked shifts. Omnichroma demonstrated greater susceptibility to degradation across all tested beverages, with substantial alterations in its polymer matrix and filler-matrix interface, likely due to its unique filler technology, which appears to enhance interactions with acidic and polyphenolic compounds. In contrast, Herculite Ultra XRV exhibited better chemical stability, showing less pronounced spectral changes, particularly in response to Coca-Cola, where only minor hydrolytic degradation was observed. These findings suggest that the structural composition of Herculite Ultra XRV, including its nanohybrid filler-matrix interactions, provides enhanced resistance to chemical degradation compared to Omnichroma. These results reinforce the role of acidic and polyphenolic agents in the chemical degradation of restorative dental materials, emphasizing the importance of understanding how common dietary exposures influence the longevity and structural integrity of composite restorations. The observed chemical interactions highlight the need for developing composite resins with enhanced resistance to acidic and staining agents to improve their long-term clinical performance.

Hedzelek et al. [19] investigated seven groups of commonly used dental materials through infrared spectroscopy within the spectral range of 4000 to 400 cm^−1^. Their study resulted in a spectral database encompassing 23 dental materials, enabling rapid identification of the chemical properties of both organic and inorganic components in dental restorative materials, such as acrylic resins and porcelains. Raman spectral peak characteristics, including frequency, frequency shifts, polarization, peak width, and intensity, provide valuable insights into the composition of the material, stress/strain states, symmetry, and orientation of inorganic filler particles, filler quality, and material quantity. Additionally, Fong et al. [20] utilized a real-time near-infrared spectroscopic approach to analyze the degree of methacrylate double-bond conversion and the polymerization rates in novel polymeric dental restorative composites.

The surface hardness test is crucial as it influences the surface properties of esthetic materials and teeth. Surface hardness is directly linked to the strength and rigidity of materials, making it a key parameter in evaluating their durability and performance [21]. The decrease in surface hardness values observed in both tested composites across different beverages can be attributed to the molecular dispersion between the matrix and filler particles within the composite resin. This process may lead to debonding at the filler-matrix interfaces, microcracking, gradual expansion, and softening, ultimately resulting in resin degradation and the dislodgement of filler particles from the resin matrix [22,23]. Supporting these findings, studies by Cabadag and Gönülol [24], as well as Kumari et al. [25], reported a similar reduction in surface hardness for bulk-fill and posterior nanocomposites, respectively, following immersion in different beverages.

The shape and size of resin composite fillers play a crucial role in determining the surface properties of restorations. This is due to the fact that the detachment of filler particles from the surface can create defects of varying sizes, depending on the particle dimensions [26,27]. In the present study, the tested composite materials featured different filler sizes, with Omnichroma consisting of uniformly arranged spherical particles measuring 260 nm.

Our study revealed that Omnichroma, a monochrome dental composite, exhibited higher resistance to surface hardness degradation compared to conventional composites under all storage conditions. This superior performance can be attributed to differences in their resin formulations. The matrix of the monochrome composite resin consists of a blend of UDMA and TEGDMA. UDMA, with its more flexible nature, has been shown to achieve a higher degree of conversion than Bis-GMA and TEGDMA, which are commonly used in conventional composites, as reported in previous studies. Since hardness is one of the most sensitive material properties influenced by the degree of conversion, these studies further demonstrated that UDMA/TEGDMA matrices achieve the highest hardness values, whereas Bis-GMA/TEGDMA matrices exhibit the lowest. The higher resistance of Omnichroma to surface hardness degradation, observed in this study, supports the growing evidence favoring monochrome composites for their enhanced mechanical properties and durability under challenging conditions. Consistent with findings by Bahgat and Hanna [23], the resin formulation plays a pivotal role, with UDMA-based matrices providing better mechanical resilience compared to traditional Bis-GMA-based composites. These results highlight the importance of optimizing resin formulations to improve the performance and longevity of dental restorations. The marked decrease in Vickers microhardness observed after red wine exposure, especially in Herculite XRV, is likely due to the combined action of acidic pH, polyphenolic compounds, and ethanol, which together promote matrix softening, filler–resin interface degradation, and plasticization of the polymer network.

Acidic beverages such as Coca-Cola exert their degrading effect on resin-based composites largely due to their phosphoric acid content, which has been shown to soften Bis-GMA monomers and compromise material stability [28]. In contexts involving wear resistance, composites may undergo characteristic failure patterns, such as delamination, where filler particles detach from the matrix, often accompanied by crack formation within the deeper layers of the material [29]. These degradation mechanisms were also evident in our scratch resistance evaluation, aligning with previous studies that observed increased delamination in materials with a higher volume of filler content [30]. The correlation between FTIR intensity loss and microhardness reduction confirms that chemical degradation at the molecular level directly influences the mechanical integrity of resin composites, with material-dependent variation in response.

However, the overall performance of restorative materials is not determined solely by filler content. Factors such as material homogeneity and the interaction between matrix and filler play a crucial role in resistance to both mechanical stress and chemical degradation [31]. In our findings, nanohybrid composites, such as Herculite XRV, exhibited enhanced resistance to surface deterioration. This behavior may be attributed to their higher filler loading and better filler distribution, which contribute to reduced water permeability and diminished hydrolysis at the resin–filler interface. Although Coca-Cola induced fewer molecular changes compared to coffee and red wine, the Raman analysis revealed localized surface erosion, likely caused by its strongly acidic pH and phosphoric acid content. This highlights that surface degradation can occur even in the absence of pronounced chemical breakdowns, underscoring the importance of integrating morphological and spectroscopic evaluations when assessing composite performance in acidic environments. Literature supports this, emphasizing that composites with uniformly distributed nano-sized fillers tend to show increased microhardness and wear resistance [32].

Conversely, the organic resin matrix—especially those based on hydrophilic monomers like Bis-GMA and TEGDMA—is more prone to water absorption and plasticization when exposed to acidic environments. This leads to weakened intermolecular bonds, reduced stiffness, and an overall decline in mechanical performance [28]. The pronounced microhardness reduction observed in our study after exposure to coffee and other low-pH beverages confirms this, particularly in materials with lower filler content. Moreover, when simulating daily exposure patterns, beverage immersion durations must reflect real-life consumption habits. Although the immersion period in this study was limited to 10 days, this protocol was based on previously validated in vitro methodologies simulating long-term cumulative exposure to dietary acids and staining agents [17]. According to Guler et al., the average person consumes approximately 3.2 cups of coffee per day. Given that a single cup is typically consumed over 15–20 min continuous 14-day immersion corresponds to nearly one year of cumulative exposure [33]. This supports the relevance of our 10-day protocol as a meaningful indicator of long-term degradation trends under realistic conditions.

In selecting the test solutions, our aim was to represent beverages with both high staining potential and varying acidity levels that are frequently encountered in daily consumption. According to the review by Paolone et al. (2022) [17], coffee, tea, and red wine are the top three most commonly used agents in laboratory protocols assessing the color stability of resin-based composites, with Coca-Cola ranking as the fourth most utilized beverage due to its low pH and erosive effect. While tea is another relevant substance, the inclusion of Coca-Cola allowed us to capture the acidic impact of a clear, pigment-free beverage, complementing the staining effects of coffee and red wine. Future studies may expand upon this by including tea or energy drinks, which also pose challenges to the longevity and esthetics of restorative materials. In line with this rationale, soda water was not included among the test solutions, as it lacks pigmentation and possesses a comparatively lower erosive potential than colored acidic beverages such as Coca-Cola. This decision is supported by prevailing trends in the literature, where soda water is seldom used in staining protocols. Instead, beverages like coffee, red wine, and cola-based drinks are more commonly selected due to their clinically relevant staining capacity and chemical aggressiveness, offering a more accurate simulation of real-life challenges faced by composite restorations.

These findings have important clinical implications for improving the longevity of composite restorations. Understanding how acidic and staining beverages affect resin materials enables clinicians to offer specific dietary and hygiene advice. Patients should be encouraged to rinse with water after consuming such drinks and maintain good oral hygiene. In high-risk cases, applying surface sealants or using composites with greater resistance to staining and hydrolytic degradation can help preserve both esthetics and durability over time.

This study expands on existing research by providing a targeted comparison between two clinically relevant resin composites—Omnichroma and Herculite XRV. Omnichroma, a single-shade composite, is recognized for its efficient shade adaptation and user-friendly application, while Herculite XRV, a nanohybrid composite, is appreciated for its durability and high polishability. Focusing on materials within the same class allows for a more controlled and clinically meaningful assessment of their performance under exposure to staining and acidic beverages. Unlike studies comparing different material types, this approach offers clearer insights into material selection for direct restorations based on real-world dietary challenges.

This study offers a comprehensive evaluation of composite resins by utilizing a multimodal approach that integrates Raman spectroscopy, FTIR analysis, and Vickers microhardness testing. This combination of methods provides valuable insights into both molecular-level changes and mechanical properties, making the findings highly relevant to clinical dentistry. Similar findings have been reported in studies using SEM–EDAX, where acidic beverages caused notable surface and compositional degradation of composite resins, with pH and titratable acidity identified as key factors. The present FTIR–Raman analysis offers complementary molecular-level insights, underscoring the importance of selecting restorative materials based on their chemical resilience in acidic environments [34]. Although SEM analysis was not included in this study, complementary surface morphology and elemental characterization of the same composites under similar beverage exposure were previously reported by our group using SEM and EDS techniques [12]. These findings support the current spectroscopic results and provide additional context regarding surface degradation mechanisms. The 10-day immersion protocol with three commonly consumed beverages closely simulates real-world dietary exposure, highlighting the importance of material selection for restoration durability. By identifying material-dependent variations in degradation, this study underscores the critical role of choosing the right composite resin to ensure long-lasting repairs, particularly in challenging oral environments.

To contextualize the present findings, Table 3 compares key parameters and outcomes from similar in vitro studies evaluating the effects of acidic beverages on dental composites. The current study stands out by integrating spectroscopic and mechanical data, offering a molecular–mechanical correlation that complements prior SEM- or hardness-based assessments.

These findings have direct clinical relevance. Omnichroma’s higher surface hardness retention suggests its suitability for posterior restorations, where mechanical stress is greatest. In contrast, Herculite XRV, showing superior chemical stability, may perform better in anterior restorations prone to staining. For patients with a high intake of acidic or pigmented beverages, clinicians should consider using surface sealants, recommending periodic polishing, or selecting materials with enhanced resistance to degradation. Such preventive strategies, alongside patient education on beverage habits and oral hygiene, can help prolong the functional and esthetic longevity of restorations.

However, several limitations of this study should be considered. As an in vitro investigation, it does not fully replicate the dynamic and multifactorial nature of the oral environment. Elements such as salivary enzymes, biofilm formation, temperature fluctuations, and mechanical stresses from chewing were not included, all of which can significantly influence the degradation and staining behavior of composite materials. Additionally, the absence of artificial saliva excludes key biochemical interactions, such as pH buffering and enzymatic activity, that could alter the response of materials to acidic or staining agents. Although distilled water was used to simulate intraoral hydration and maintain specimen consistency, this choice was primarily made to avoid the biochemical complexity and variability introduced by artificial saliva, such as enzymatic activity, mineral content, and buffering effects. These factors could interfere with the isolated evaluation of chemical degradation caused specifically by the staining beverages. The decision aligns with previous in vitro protocols [17] that prioritize experimental control when assessing direct material–beverage interactions. While the use of pure beverages allowed for the isolation of beverage-specific effects on chemical degradation and surface hardness, it does not fully replicate the complex oral environment, where saliva plays a crucial buffering and protective role. Incorporating artificial saliva or saliva-beverage mixtures could better simulate in vivo conditions by introducing enzymatic, ionic, and pH-regulating effects. However, to minimize confounding variables and ensure reproducibility, we opted for direct exposure to undiluted beverages.

The study also did not apply thermocycling or artificial aging protocols, which are commonly used to simulate thermal and mechanical fatigue over time. This deliberate omission allowed us to isolate and analyze the specific chemical effects of beverage exposure on resin composites without interference from thermal or mechanical aging factors, thereby enhancing experimental clarity and internal validity. The 10-day immersion period, while useful for short-term evaluation, may not accurately represent long-term clinical exposure. Furthermore, the analysis was limited to two composite resins, which, although selected for their clinical relevance and contrasting compositions, may not reflect the behavior of other resin systems such as bulk-fill, nanohybrid, or bioactive composites. Although this study employed paired *t*-tests to assess within-group differences in microhardness before and after immersion, future research involving larger sample sizes and more complex comparisons may benefit from the use of ANOVA and post hoc analyses to further explore intergroup interactions and material-specific responses to dietary challenges. Lastly, only three beverages were tested, excluding other commonly consumed acidic drinks like fruit juices or energy drinks that may also impact restorative materials. Future studies should aim to incorporate a wider range of materials, longer testing durations, simulated oral conditions, and more diverse dietary agents to provide a deeper understanding of composite performance under realistic clinical challenges.

## 5. Conclusions

This study demonstrates that commonly consumed dietary beverages—coffee, red wine, and Coca-Cola—have a measurable impact on the chemical composition and surface hardness of dental composite resins. FTIR and Raman spectroscopy revealed that coffee and red wine induced the most substantial molecular degradation, particularly in functional groups such as carbonyl (C=O), aromatic (C=C), and siloxane (Si–O–Si). Among the materials tested, Omnichroma exhibited more pronounced spectroscopic changes, indicating higher chemical reactivity, while Herculite XRV showed greater structural stability under similar conditions.

Despite this, Vickers microhardness results indicated that Omnichroma retained superior surface hardness, with the smallest reduction observed after Coca-Cola exposure. In contrast, Herculite XRV experienced a significant hardness drop, especially after immersion in red wine. These findings reflect the complex interaction between composite formulation, beverage composition, and degradation behavior.

Overall, this research underscores the importance of selecting restorative materials based not only on mechanical strength or esthetics but also on their resistance to real-life dietary challenges. Clinically, Omnichroma may be preferred in stress-bearing posterior restorations due to its mechanical resilience, while Herculite XRV may offer advantages in anterior restorations exposed to staining agents, thanks to its superior chemical stability. Additionally, practitioners should consider recommending dietary moderation, enhanced oral hygiene, or the use of surface sealants for patients with a high intake of acidic or pigmented beverages. These findings support a more personalized, preventive, and evidence-based approach to restorative material selection and treatment planning.

## Figures and Tables

**Table 1:** Specifications of the evaluated composite resins: composition, manufacturer, and material properties.

Product Name	Type	Resin Matrix	Lot Number	Manufacturer
Herculite XRV	Nanohybrid resin composite	Bis-GMA, TEGDMA Fillers: 78% wt, 59% vol	10198512	Kerr, Scafati, Italy
Omnichroma	Suprananospherical composite	UDMA, TEGDMA Fillers: 79% by weight; filler (SiO_2_-ZrO_2_ 260 nm)	123E83	Tokuyama-Dental, Tokyo, Japan

**Table 2:** Descriptive statistics of Vickers microhardness for Herculite XRV and Omnichroma composites under different conditions.

Product Name		Mean (Standard Deviation)	Skewness	Kurtosis
Herculite XRV	Control	34.10 (±1.53)	0.01	−1.14
	Red Wine	22.45 (±0.58)	0.91	1.92
	Coffee	27.35 (±0.84)	0.31	−0.68
	Coca-Cola	28.60 (±0.73)	−0.18	−1.58
Omnichroma	Control	33.20 (±0.84)	0.07	−0.52
	Red Wine	29.60 (±0.96)	0.98	0.23
	Coffee	27.42 (±1.14)	0.79	−0.20
	Coca-Cola	30.26 (±1.30)	−0.00	−1.34

**Table 3:** Summary of analytical approaches and findings from beverage-exposure studies on dental composites.

Study	Composites Evaluated	Analytical Methods	Immersion Solutions	Key Findings
Current study	Herculite XRV, Omnichroma	FTIR, Raman, Vickers hardness	Coffee, red wine, Coca-Cola	Chemical degradation correlates with hardness loss; Omnichroma more resistant to mechanical change
Gradinaru et al. (2023) [34]	3 commercial composites (microhybrid)	SEM, EDAX, color, and morphology	Coffee, red wine, Coca-Cola, tea, energy drinks	Surface damage correlates with beverage acidity and staining potential; microhybrids more vulnerable
Kedici Alp et al. (2023) [10]	2 universal single-shade composites	Surface roughness, SEM	Tea, coffee, wine, orange juice	Color and texture degradation linked to beverage type; tea and wine most aggressive
Das et al. (2024) [32]	Bulk-fill and nanohybrid composites	Surface microhardness, SEM	Acidic beverages	Significant hardness loss and surface erosion observed after acidic exposure
Bahgat & Hanna (2024) [23]	Monochrome vs. conventional composites	Surface hardness in food simulants	Coffee, tea, cola	Monochrome showed better hardness retention than conventional composite

## Data Availability

The data presented in this study are available on request from the corresponding author.

## References

[1] Balhaddad A.A., Kansara A.A., Hidan D., Weir M.D., Xu H.H.K., Melo M.A.S. (2019). Toward Dental Caries: Exploring Nanoparticle-Based Platforms and Calcium Phosphate Compounds for Dental Restorative Materials. Bioact. Mater..

[2] Iordache S.-M., Iordache A.-M., Gatin D.I., Grigorescu C.E.A., Ilici R.R., Luculescu C.-R., Gatin E. (2024). Performance Assessment of Three Similar Dental Restorative Composite Materials via Raman Spectroscopy Supported by Complementary Methods Such as Hardness and Density Measurements. Polymers.

[3] Ferracane J.L. (2024). A Historical Perspective on Dental Composite Restorative Materials. J. Funct. Biomater..

[4] De Abreu J.L.B., Sampaio C.S., Benalcázar Jalkh E.B., Hirata R. (2021). Analysis of the Color Matching of Universal Resin Composites in Anterior Restorations. J. Esthet. Restor. Dent..

[5] Yadav R., Singh M., Meena A., Lee S.-Y., Park S.-J. (2023). Selection and Ranking of Dental Restorative Composite Materials Using Hybrid Entropy-VIKOR Method: An Application of MCDM Technique. J. Mech. Behav. Biomed. Mater..

[6] Yadav R., Saini S., Sonwal S., Meena A., Huh Y.S., Brambilla E., Ionescu A.C. (2024). Optimization and Ranking of Dental Restorative Composites by ENTROPY—VIKOR and VIKOR—MATLAB. Polym. Adv. Techs..

[7] Khan A.S., Khalid H., Sarfraz Z., Khan M., Iqbal J., Muhammad N., Fareed M.A., Rehman I.U. (2017). Vibrational Spectroscopy of Selective Dental Restorative Materials. Appl. Spectrosc. Rev..

[8] Ramakrishnaiah R., Rehman G.U., Basavarajappa S., Al Khuraif A.A., Durgesh B.H., Khan A.S., Rehman I.U. (2015). Applications of Raman Spectroscopy in Dentistry: Analysis of Tooth Structure. Appl. Spectrosc. Rev..

[9] Otel I. (2023). Overall Review on Recent Applications of Raman Spectroscopy Technique in Dentistry. QuBS.

[10] Kedici Alp C., Arslandaş Dinçtürk B., Altınışık H. (2023). The Effect of Food-Simulating Liquids on Surface Features of Single-Shade Universal Composites: An in Vitro Study. J. Int. Soc. Prev. Community Dent..

[11] Hajdu A.I., Dumitrescu R., Balean O., Lalescu D.V., Buzatu B.L.R., Bolchis V., Floare L., Utu D., Jumanca D., Galuscan A. (2024). Enhancing Esthetics in Direct Dental Resin Composite: Investigating Surface Roughness and Color Stability. J. Funct. Biomater..

[12] Hajdu A.I., Dumitrescu R., Balean O., Jumanca D., Sava-Rosianu R., Floare L., Bolchis V., Vlase T., Galuscan A. (2024). Microscopic and Color Changes in Direct Dental Restorative Composite Resins upon Immersion in Beverages: Characterization by Scanning Electron Microscopy (SEM) and Energy-Dispersive X-Ray Spectroscopy (EDS). Biomedicines.

[13] Nair K.C., Dathan P.C., Sreeba S.B., Soman A.K. (2022). Hardness of Dental Materials Is an Essential Property That Determines the Life of Restorations—An Overview. Acta Sci. Dent. Sci..

[14] HerculiteTM Ultra. https://www.kerrdental.com/kerr-restoratives/herculite-ultra-universal-nanohybrid-dental-composite.

[15] OMNICHROMA Composite—Tokuyama Dental America. https://www.tokuyama-us.com/omnichroma-dental-composite/.

[16] Gönülol N., Yılmaz F. (2012). The Effects of Finishing and Polishing Techniques on Surface Roughness and Color Stability of Nanocomposites. J. Dent..

[17] Paolone G., Formiga S., De Palma F., Abbruzzese L., Chirico L., Scolavino S., Goracci C., Cantatore G., Vichi A. (2022). Color Stability of Resin-based Composites: Staining Procedures with Liquids—A Narrative Review. J. Esthet. Restor. Dent..

[18] Şerban V.A., Răduţă A. (2014). Ştiinţa şi Ingineria Materialelor.

[19] Hędzelek W., Marcinkowska A., Domka L., Wachowiak R. (2008). Infrared Spectroscopic Identification of Chosen Dental Materials and Natural Teeth. Acta Phys. Pol. A.

[20] Fong H., Dickens S., Flaim G. (2005). Evaluation of Dental Restorative Composites Containing Polyhedral Oligomeric Silsesquioxane Methacrylate. Dent. Mater..

[21] Al-Samadani K.H. (2016). Surface Hardness of Dental Composite Resin Restorations in Response to Preventive Agents. J. Contemp. Dent. Pract..

[22] Szczesio-Wlodarczyk A., Sokolowski J., Kleczewska J., Bociong K. (2020). Ageing of Dental Composites Based on Methacrylate Resins—A Critical Review of the Causes and Method of Assessment. Polymers.

[23] Bahgat H.A., Hanna N.M.A. (2024). Comparative Evaluation of the Surface Hardness of Monochrome Composite and Conventional Composite after Immersion in Food-Simulating Liquids: An In Vitro Study. Int. J. Prosthodont. Restor. Dent..

[24] Cabadag Ö.G., Gönülol N. (2021). The Effects of Food-Simulating Liquids on Surface Roughness, Surface Hardness, and Solubility of Bulk-Fill Composites. J. Adv. Oral Res..

[25] Kumari C.M., Bhat K.M., Bansal R., Singh N., Anupama A., Lavanya T. (2019). Evaluation of Surface Roughness and Hardness of Newer Nanoposterior Composite Resins after Immersion in Food-Simulating Liquids. Contemp. Clin. Dent..

[26] Sang E.J., Song J.-S., Chung S.H., Jin B.-H., Hyun H.-K. (2021). Influence of a New Polishing System on Changes in Gloss and Surface Roughness of Resin Composites after Polishing and Brushing. Dent. Mater. J..

[27] Guler S., Unal M. (2018). The Evaluation of Color and Surface Roughness Changes in Resin Based Restorative Materials with Different Contents After Waiting in Various Liquids: An SEM and AFM Study. Microsc. Res. Tech..

[28] Al-Angari S.S., Eckert G.J., Sabrah A.H.A. (2021). Color Stability, Roughness, and Microhardness of Enamel and Composites Submitted to Staining/Bleaching Cycles. Saudi Dent. J..

[29] Fan H.-Y., Gan X.-Q., Liu Y., Zhu Z.-L., Yu H.-Y. (2014). The Nanomechanical and Tribological Properties of Restorative Dental Composites after Exposure in Different Types of Media. J. Nanomater..

[30] Murase Y., Kotake H., Kusakabe S., Okuyama K., Tamaki Y., Hotta M. (2020). Use of New Scratch Test and Tensile Test for Evaluation of Bond Strength of Selfadhesive Flowable Resin Composite for Repair of Artificial Tooth Erosion. Dent. Mater. J..

[31] Paolone G., Pavan F., Mandurino M., Baldani S., Guglielmi P.C., Scotti N., Cantatore G., Vichi A. (2023). Color Stability of Resin-based Composites Exposed to Smoke. A Systematic Review. J. Esthet. Restor. Dent..

[32] Das K., Murthy C.S., Naganath M., Mehta D., Anitha Kumari R., Karobari M.I., Venkataiah V.S. (2024). Insights Into the Effects and Implications of Acidic Beverages on Resin Composite Materials in Dental Restorations: An In Vitro Study. J. Esthet. Restor. Dent..

[33] Guler A.U., Yilmaz F., Kulunk T., Guler E., Kurt S. (2005). Effects of Different Drinks on Stainability of Resin Composite Provisional Restorative Materials. J. Prosthet. Dent..

[34] Gradinaru I., Vasiliu A.L., Bargan A., Checherita L.E., Ciubotaru B.-I., Armencia A.O., Istrate B., Dascalu C.G., Antohe M.E. (2023). The Influence of Beverages on Resin Composites: An In Vitro Study. Biomedicines.

